# Sustainable Development Approaches through Wooden Adhesive Joints Design

**DOI:** 10.3390/polym15010089

**Published:** 2022-12-26

**Authors:** Catarina S. P. Borges, Shahin Jalali, Panayiotis Tsokanas, Eduardo A. S. Marques, Ricardo J. C. Carbas, Lucas F. M. da Silva

**Affiliations:** 1 Instituto de Ciência e Inovação em Engenharia Mecânica e Engenharia Industrial (INEGI), Campus da FEUP, R. Dr. Roberto Frias, 4200-465 Porto, Portugal; 2 Departamento de Engenharia Mecânica, Faculdade de Engenharia (FEUP), Universidade do Porto, R. Dr. Roberto Frias, 4200-465 Porto, Portugal

**Keywords:** sustainable development, wood adhesive joint, bio-adhesive, literature review, mechanical behaviour

## Abstract

Over recent decades, the need to comply with environmental standards has become a concern in many industrial sectors. As a result, manufacturers have increased their use of eco-friendly, recycled, recyclable, and, overall, more sustainable materials and industrial techniques. One technique highly dependent on petroleum-based products, and at the edge of a paradigm change, is adhesive bonding. Adhesive bonding is often used to join composite materials and depends upon an adhesive to achieve the connection. However, the matrices of the composite materials and the adhesives used, as well as, in some cases, the composite fibres, are manufactured from petrochemical products. Efforts to use natural composites and adhesives are therefore ongoing. One composite that has proven to be promising is wood due to its high strength and stiffness (particularly when it is densified), formability, and durability. However, wood must be very carefully characterised since its properties can be variable, depending on the slope of the grains, irregularities (such as knots, shakes, or splits), and on the location and climate of each individual tree. Therefore, in addition to neat wood, wood composites may also be a promising option to increase sustainability, with more predictable properties. To bond wood or wooden composite substrates, bio-adhesives can be considered. These adhesives are now formulated with increasingly enhanced mechanical properties and are becoming promising alternatives at the structural application level. In this paper, wooden adhesive joints are surveyed considering bio-adhesives and wood-based substrates, taking into consideration the recent approaches to improve these base materials, accurately characterise them, and implement them in adhesive joints.

## 1. Introduction

Polymeric products are used in a wide range of applications, including non-structural applications, such as plastic bottles, bags, and several household items, and structural applications in several industries, such as automotive, aerospace, and naval. Structurally, these materials are rarely used in their pure form but as matrices of composite materials, such as carbon fibre-reinforced polymers (CFRPs) and glass fibre-reinforced polymers (GFRPs). Composites are usually notch-sensitive, and any joining using bolts or rivets, which require the creation of holes in the material, should therefore be avoided. Joining through welding can also damage the material due to local heating. Adhesive joining has become an intriguing alternative technique for these applications since it does not damage the materials to be bonded (substrates). At the same time, adhesive bonding creates a more uniform stress distribution in the connection area [1]. However, it should be noted that most of the structural adhesives used today still consist of petroleum-based materials [2].

Due to their low cost, durability, low weight, versatility, and simpler manufacturing processes, these polymeric products are used to replace other materials, such as metals, in many applications. As a result, they are causing environmental hazards [2]. One of the most significant issues with polymers is their disposal as such materials require extremely long times to degrade. In more developed countries, plastics are used for energy recovery. However, the combustion of these materials, mainly those containing halogens, contributes to the emission of polluting gases, and of carbon dioxide in particular [3,4].

To reduce the impact of fossil-based products, there has been an effort lately to develop and adopt bio-based materials [5,6]. In the manufacture of eco-friendly load-bearing structures, tree- and plant-based products can play an important role, for instance, by enabling the manufacture of composites made from flax, jute, palm, and other natural fibres. Focusing on trees, throughout history, wood has been used for various daily purposes because it is a naturally renewable resource [7]. Its flexibility to be cut into different shapes, along with its durability, wear and environmental resistance, mechanical strength, low weight, abundance in many locations around the world, and reasonable cost, make it an attractive material. Many artefacts of refined geometrical configuration have also been developed from wood, taking advantage of its ability to change form under specific environmental conditions (i.e. temperature, loading rate, and moisture content) [8].

Wood is considered a natural composite, and, as with most composites, it is sensitive to the creation of holes and other notches. To join wood parts, therefore, classical joining methods such as riveting and bolting would be unsuitable. For these materials, adhesive bonding appears to be a well-suited joining technique since it does not introduce stress concentrations and creates a larger and more uniform bonded area. Adhesive joints often work in shear to maximise the resistance area of the material. However, if two substrates are overlapped, bonded, and loaded in opposite directions, the loads applied will tend to align, creating a flexural moment at the ends of the overlap. Therefore, the stress distribution along the adhesive is not purely in shear but a combination between shear and tensile (peel). Accordingly, the failure load and failure mode of the joint will depend on the combined properties of the adhesive and the substrates. In an adhesive joint between wood substrates, this contribution of the peel stress may lead to delamination between different grain plies and, thus, the complete failure of the joint. To prevent this failure mode, recent techniques, such as densification, substrate toughening, or the physical modification of the adhesive, have been proposed.

With this in mind, the use of adhesives to bond wood substrates requires special attention to design efficient, safe, and high-performance wooden structures. Moving towards the advanced use of wooden structures (for example, in vehicles), different physical and chemical modifications have been proposed to enhance the mechanical performance of these base materials. Additionally, the accurate characterisation and standardisation of the natural materials used are required for a reliable and repeatable structure.

In this paper, bio-adhesives that can be used in wooden joints will be discussed first, followed by wood substrates, also exploring how to characterise them and tune their mechanical properties to highly demanding applications. Next, wood/bio-adhesive joints will be analysed, discussing their properties, testing procedures, and durability. The paper closes with a justification of concluding remarks.

## 2. Bio-Adhesives

Structural adhesives used in industrial applications are often derived from petroleum. These adhesives, although having advanced mechanical and physical properties, present important disadvantages from an economic and environmental point of view [9,10]. Economically, petroleum is increasingly seen as a risky supply source due to the unpredictability of the petrochemical industry, which may lead to a sudden increase in the price of the material, reflected in the production costs of the adhesive. Environmentally, fossil-based products influence the health of living species and contribute to air, soil, and water pollution [9,11,12,13]. Therefore, an increased requirement of industries and manufacturers to produce and use bio-based adhesives obtained from different natural resources is observed, aiming to decrease the harmful effects of fossil-based petroleum products.

To avoid possible reader confusion, we will now distinguish a bio-based polymer from a biodegradable polymer. A bio-based polymer refers to a product obtained from natural renewable resources such as trees, fruit, or animals. The advancement of bio-based adhesives can result in the production of high-quality adhesives with considerable humidity and chemical resistance and high adhesion properties, which are good alternatives to fossil-based adhesives. In contrast, biodegradable polymers are those that are capable of degrading naturally. These polymers are highly prone to mechanical and chemical degradation due to, for instance, water, UV radiation, and time. Biodegradable polymers are thus not a suitable suggestion for structural applications [14].

### 2.1. Bio-Adhesive Families

**Figure 1:**
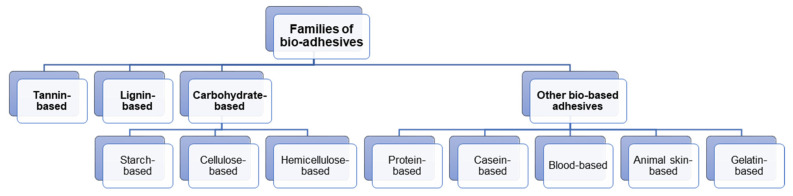
The most significant families of bio-adhesives discussed in this paper.

To develop more eco-friendly adhesive joints, several structural bio-adhesives have been produced (Figure 1). In parallel, many bio-based polymers, which are natural, renewable, and non-petroleum-based, are being adapted and characterised to be used in adhesive applications. There are only a limited number of materials that can be used to fulfil this role. Although natural materials are used to produce bio-based adhesives, the modifications that can be made and the obtained properties are always naturally restricted by the base material [15,16,17].

Some materials that have been explored as natural adhesives with satisfactory results are tannin, lignin, carbohydrates, unsaturated oils, proteins, and protein hydrolysates. Dissolved wood and wood welding by self-adhesion have also been presented as alternatives to bonding.

#### 2.1.1. Tannin-Based Adhesives

Tannin is among the first natural materials to be used as a bio-adhesive in modern years, being used in some countries since the 1970s [18]. Tannin molecules are inexpensive and widely available, making them suitable as natural polymer replacements. These molecules can be found in trees, mainly in their soft tissues, such as leaves, needles, or bark [19]. Tannin is traditionally used in leather industries to transform hide into leather. In adhesives, tannins are used as a substitute for phenols.

Tannins are generally divided into two groups: hydrolysable and non-hydrolysable (or condensed). The first group is less attractive from both a chemical and economic viewpoint because these tannins, in their natural state, lack molecular structure, can only substitute a low amount of phenol, have low nucleophilicity, and have a smaller production. Condensed tannins, therefore, represent the majority of tannin production [16,19].

In the quest to improve the use of tannins for industrial applications, two main routes for tannin hardening can be identified: hardening using hexamethylene hardener and hardening by auto-condensation. The hexamethylene–tannin adhesives demonstrated very low emissions, with the emissions almost limited to those generated by wood heating. Additionally, the manufactured wood panels would meet the standards for interior and exterior use [16]. Tannin hardening by auto-condensation was also reported to be effective, particularly for interior-grade particle boards. In contrast, it has the limitation of generally requiring long pressing times, even though the tannin of the pecan nut was found to require short pressing times, matching industrial standards [20]. Both approaches for tannin hardening are being considered by industrial groups for practical applications [16].

#### 2.1.2. Lignin-Based Adhesives

Lignin is one of the most abundant compounds in biomass after cellulose and hemicellulose [21]. This material is the natural glue in trees and plants such as wheat or oats, and it constitutes an attractive replacement for conventional adhesives used in wood adhesive joints. Lignin is a phenolic material, the major by-product of the paper pulping process [22,23]. Therefore, due to its availability and low cost, there is interest in taking advantage of its material properties in the adhesive industry.

The most prominent objective of the use of lignin and lignosulphonates is their incorporation into phenolic adhesives for wood panels, such as urea–formaldehyde (UF) and phenol–formaldehyde (PF) [22,24,25]. Incorporating lignin into these adhesives can decrease their cost and environmental footprint. Regarding PF resins, it has been seen that lignin from softwood is more desirable for synthesising these resins than lignin from hardwood or agricultural waste. After comparing different types of lignin, Tejado et al. [26] concluded that kraft lignin presents the best compatibility to replace phenol. Contrarily, lignin has a low level of reactive sites and reactivity; therefore, the advantage in terms of cost is somehow lost in the increasing panel pressing time [16]. An alternative to the industrial use of lignin is to pre-react it with formaldehyde, forming methylated lignin. This methylated lignin is then added to PF resins. This alternative has been used for plywood mills with good results since the low pressing time is not a requirement in this application [27].

Research does exist on the use of pure lignin-based adhesives, but because of their unsuitability for commercial production due to their lengthy press times or the fact that they are highly corrosive for certain equipment, they have never been used in industrial applications. Just one alternative is used today, which is based on the self-coagulation and cross-linking of lignin in the presence of a mineral acid using aluminium salt catalysts [28,29,30,31].

In 2008, the Environmental Protection Agency (EPA) classified formaldehyde as a carcinogenic substance, forcing its removal from consumer products [32]. In the scope of the changes suggested to make this transition in regards to the formulation of adhesives, lignin has been proposed as an eco-friendly alternative to formaldehyde [32]. To this end, Geng and Li [33] proposed a new adhesive formulation using kraft lignin and polyethyleneimines. This adhesive was used to manufacture plywood, and it was seen that higher strength and water resistance were achieved when the mass content of lignin was double the mass content of polyethylenimine. Therefore, it can be seen that new formaldehyde-free, eco-friendly adhesive formulations using lignin are also being developed.

#### 2.1.3. Carbohydrate-Based Adhesives

Carbohydrates are substances containing carbon, hydrogen, and oxygen [28]. This compound family comprises all substances with the general formula C_x_(H_2_O)_y_, with the subscripts *x* and *y* being equal to three or higher. In these compounds, one carbon forms a carbonyl group, which can be an aldehyde group or a ketone group, and the second one forms a hydroxyl group [28,34]. Therefore, carbohydrates are polyhydroxy aldehydes or polyhydroxy ketones. These materials can be applied directly to wood or can be used to modify existing PF and UF adhesives or form degradation compounds used as adhesive building blocks [16,35].

Some of the most commonly used carbohydrates for wood bonding are starch-based, cellulose-based, and hemicellulose-based carbohydrates [28].

Starch is a biopolymer widely used in binders, sizing materials, adhesives, and pastes [36]. This biopolymer comprehends glucose units, bonded by glycosidic bonds, and it is relevant for adhesive use since it has good adhesion and good film formation properties, along with its availability in nature and low cost [28,37,38]. However, starch is prone to hydrogen bonding and therefore has low durability in humid conditions, meaning that certain modifications are required before this material can be considered as a component of a bio-based adhesive. These modifications can aim to increase the shelf life [39], water resistance and hydrophobicity [39,40], mechanical properties [41,42], and dimensional stability [43] of the final bonded structure. Recently, Sulaiman et al. [44] analysed starch as an additive to a UF adhesive used for particle board manufacturing. Although the final product met industrial standards in terms of strength, the water resistance exhibited was lower. This shows that there is still room to improve in terms of formulation before these adhesives have a wider market implementation.

Cellulose is found in trees, plants, algae, and oomycetes, making it the most common organic compound on Earth [45]. The most appealing mechanical properties of this material for adhesive use are its high compressive and tensile strength. A dialdehyde cellulose derivative was proposed as an adhesive in [46], and it showed high bonding strength, even higher than the wood itself, leading to delamination in the wood. This evidences the high potential of this material for wood bonding. Cellulose nanocrystals were used in a soap-free emulsion polymerisation of vinyl acetate, producing a good waterborne wood adhesive. Cellulose nanofibrils have been proposed as a UF adhesive filler [47,48], showing that joint strength can be increased with the addition of small amounts of filler and that the optimum cellulose content should be evaluated for each base material and cellulose filler used.

Hemicellulose, as with cellulose, is found in the cell walls of trees and plants, but it has less commercial value than the starch-based and cellulose-based carbohydrate adhesives discussed above [28,49]. Although hemicellulose is a promising type of bio-adhesive, particularly with ammonium zirconium (IV) carbonate cross-linkers [50], the batch-to-batch variation of properties (e.g. molecular weight) was suspected to influence bond strength. Indeed, this suspicion was eventually verified in [51] using a nonlinear relation.

#### 2.1.4. Other Bio-Based Adhesives

Other bio-adhesives for wood applications exist, including protein-based, casein-based, blood-based, animal-skin-based, and gelatine-based adhesives. Among these, the most relevant are the protein-based wood adhesives.

Proteins consist of amino acids and are the basis of living species [28]. Although the development of protein-based adhesives is being pursued and thus far appear promising, they are not yet compatible with industrial requirements [16,28,52]. More specifically, they are still inconsistent in terms of composition and mechanical properties [28]; they are expensive when compared to classical adhesives; and they present a short pot life, limited supply, and low resistance to humidity [36].

For instance, soy protein adhesives have been proposed for joining wood panels with good results [37,38], even though this would be impossible without extensive modifications. The modification processes of soy protein often focus on improving the mechanical behaviour of the adhesive, which can be achieved via thermal treatments [39], microwave treatments [40], modifications in the cross-linking [41,42,53], or biomimetic modification [43], among other aspects. Nevertheless, there are other concerns that need to be tackled, such as the ability of proteins to penetrate wood voids since they are usually significantly large molecules with high molecular weights. If the adhesive does not penetrate the wood substrate, its ability to form strong bonds through mechanical interlocking is lost, leading to the adhesive failure of the joint.

Another option under development is the use of water-soluble soybean polysaccharide, a by-product of soy after the protein and oil are extracted [52]. Since this material has been used in packaging films due to its good formability and adhesive properties, it may be a viable path for developing bio-adhesives. However, this material still needs to be able to create a strongly cross-linked structure before it can be considered for adhesive use [45,46].

Kowaluk et al. [54] studied the shear strength and in-wood damage ratio of wood bonded with bio-adhesives such as gelatine, casein, gluten, and polyvinyl acetate. The highest shear strength was achieved for a gelatine/acetic acid mixture.

### 2.2. Durability of Bio-Adhesives

As already mentioned, one of the main concerns regarding bio-adhesives is their durability under severe conditions such as humidity, high temperature, and long operational times [14,55]. This behaviour is dependent on each adhesive and any additives used, and this topic is thus far relatively unexplored for bio-adhesives. However, to date, several studies regarding bio-adhesives or a combination of bio-based and petroleum-based adhesives have been conducted in this area.

Environmental humidity, for instance, was pointed out as a particularly concerning aspect of soy-based adhesives. Tian et al. [47] tried to tackle this issue for soy protein films by mixing polyurethane (PUR) or a natural rubber into the film. Humidity studies are usually conducted by subjecting a thin plate of material to a given humidity condition and evaluating its mass change. However, this procedure proved unsuitable for these films. Instead, the hydrophilicity was evaluated by measuring the contact angle of the film surface, concluding that the addition of PUR or a natural rubber may contribute to making the material more hydrophobic and, therefore, more resistant to moisture. The thermal stability of soy-based materials was also seen to improve by adding inorganic fillers [49,50].

Altuna et al. [51] analysed epoxy resins cured with diglyceryl ether of Bisphenol A (DGEBA) and soybean oil (ESO). The authors concluded that as the ESO content increases, the storage modulus and the *T*_g_ of the adhesive decrease. For 100% ESO, the *T*_g_ is close to half of the *T*_g_ with 0% ESO [51]. Therefore, it can be seen that adding ESO can approximate the temperature at which the material is in its rubbery state to room temperature, which can inhibit its use in a wide range of applications that require a stronger and stiffer material.

Baumberger et al. [56] developed films by extrusion followed by thermal moulding containing up to 30% kraft lignin combined with starch. The mechanical properties of the composite were evaluated through tensile tests at two humidity levels. Lignin offered an increase in strength and strain-to-failure for 58% relative humidity (RH) and contents up to 20%. However, at 71% RH, lignin has the opposite effect from 0 to 30%. It was seen that lignin decreased the affinity of the films to water, and this behaviour was attributed to a mixture of the hydrophilic starch matrix and hydrophobic lignin. This is a matter of concern in adhesives with additives since the adhesive matrix is usually more hydrophilic than the reinforcement, which makes the adhesive/reinforcement interface a preferential path for water diffusion [57].

In summary, although bio-adhesives have certain advantages, such as their low cost, environmental impact, and availability of the source material, and although their mechanical properties are constantly being improved, their durability remains an issue. Moreover, although efforts are being made to improve the durability of bio-adhesives, characterising durability under different humidity conditions, temperatures, and loading conditions (e.g., quasi-static, fatigue, impact, creep) is an area which calls for further research.

## 3. Wood Substrates

Metals and composites are commonly used as substrates for structural adhesive joints thanks to their essential advantages, such as high load-bearing capacity, impact resistance, and high reparability. Nonetheless, these materials pose environmental concerns that endanger human health.

Consequently, eco-friendly materials adopted from natural resources have attracted the attention of researchers and engineers for the development of bio-substrates. Wood and bio-composites are among the most successful load-bearing structures, which have been used for some time in the civil engineering sector [58] and are now intended for future applications in different high-performance structures in, for example, the automotive and aerospace industries, in seeking to develop lighter, safer, and cheaper structural components. Wood, in particular, possesses satisfactory mechanical properties and is readily available all over the world. Consequently, many research groups have been working over recent decades on characterising the mechanical and fracture toughness properties of wood, as well as developing damage detection techniques for wood. However, the complex structure of wood poses challenges in the mechanical characterisation of the substrates [59], as detailed below.

### 3.1. Wood and Wood Products

**Figure 2:**
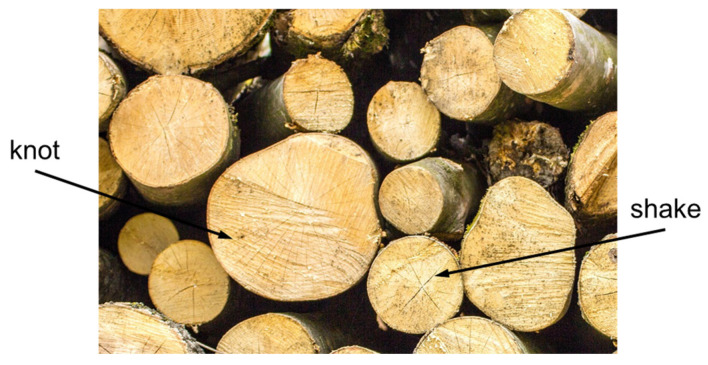
Cross-sections in wood that indicate two common defects.

Due to their advantages, wooden substrates have become one of the most frequently used bio-based materials for load-bearing structures [60]. The mechanical behaviour of wood is a function of the direction and the size of its fibres (grains), and of the extent and size of natural defects, such as knots and shakes, according to the biological source of the wood (Figure 2). The natural imperfections in the structure of wood can be the reason for premature structural failure. Nonetheless, several ways to minimise the effects of those imperfections have been proposed, such as wood densification, using wood particles or laminated wood, and using wooden composites. These advances have increased the usage of wood-based materials for structural applications in recent decades [61,62].

The roadmap to producing efficient wood products can be aided by some simplifying assumptions. Wood composite laminates can be thought of as a specific type of adhesive joint with different substrate sizes from the grain size to particles. Under this assumption, the mechanical behaviour of wooden composite laminates is greatly simplified by considering the macroscale behaviour of the adhesive bond [60]. This assumption divides advances in composite laminates into three main groups according to their functions: improvement in the mechanical behaviour of the wooden substrate, advancements in the adhesive properties and bonding behaviour, and techniques to improve the bonding design of laminates. Examples of the advances for each group are reviewed below.

One approach to improving the mechanical behaviour of wood substrates is the densification of wood [14,63]. An increase in the number of cross-links in the adhesive structures could enhance the adhesive behaviour by increasing the adhesive stiffness and improving the water resistance characteristics of the adhesive using mechanical treatments or additives. In general, the development of composite design by changing the layup and manufacturing process is the most effective way to improve the mechanical performance of wood composite structures.

Another method that has been shown to improve the mechanical performance of a wooden composite is the use of a toughening layer to reduce the peel stress at the edges and prevent composite delamination by reducing stress transfer through the thickness of the composite. The behaviour of a wooden composite is a function of the physical and mechanical behaviour of wood, such as wettability, compatibility, good adhesion, and mechanical interlocking. Improving these parameters promises a stronger adhesion between the resin and the composite particles. The geometries of wood particles, able to avoid stress concentrations, have a significant effect on the strength of the entire structure [64].

### 3.2. Mechanical Characterisation of Wood

**Figure 3:**
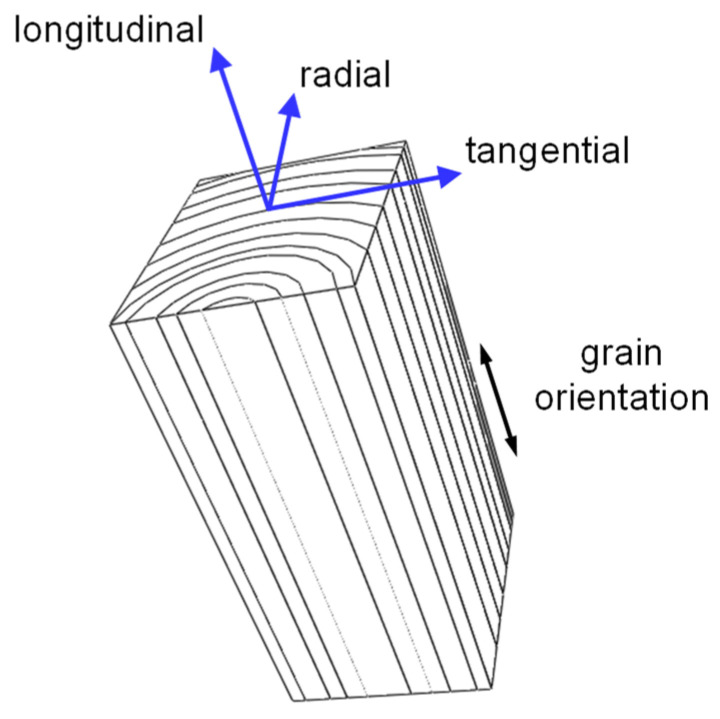
Orthotropic directions of wood.

The natural structure of wood makes its mechanical behaviour anisotropic. This anisotropic behaviour occurs because of wood cells that are oriented parallel to the grain direction. Due to the circular grain direction of wood and tree trunks, a cylindrical coordinate system is normally used in the analysis of the mechanical and physical properties of wood [60,65]. This coordinate system consists of the longitudinal (L), radial (R), and tangential (T) axes, as shown in Figure 3. The elastic response of wood is described by nine independent elastic constants (based on the generalised Hooke’s law [66]), which must be obtained through mechanical experiments, as with an anisotropic composite laminate.

Wood cells are oblong and predominantly oriented in the grain direction, giving rise to a strong direction along the longitudinal axis of wood. Thus, if the external load is applied in the grain direction, wood shows its highest strength, as also happens with a typical fibre-reinforced composite ply loaded in the fibre direction. The strength and stiffness of wood remarkably decrease in the tangential and radial directions. Therefore, the loading directions of a wood structure should be seriously considered during its design [60,67].

According to the significantly higher strength and stiffness of wood in the grain direction, the cellular structure is compressed in its weakest directions, revealing similar property values, currently designated as properties perpendicular to the grain. Consequently, this material usually fails in the form of wood splitting along planes parallel to the grains, often due to shear or tension perpendicular to the grain.

Different experimental tests are suggested to determine the elastic and strength properties of wood. In particular, tensile and compressive tests have been introduced to estimate the Young’s moduli of wood, considering the grain orientation.

**Figure 4:**
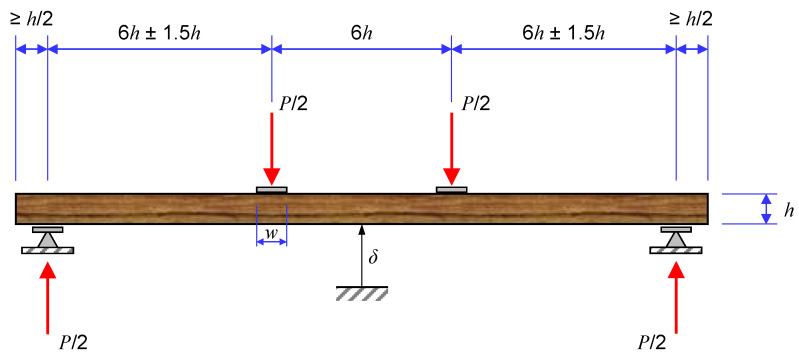
A scheme of the four-point bending test configuration for determining the shear properties of wood.

In tensile tests of wood specimens, the use of steel end-tabs is mandatory to avoid stress concentration and specimen failure in the grips [68]. Given the viscoelastic behaviour of wood, the strain rate under displacement control condition should be approximately 0.00005/s to determine the static properties of wood. A more complex procedure is required to determine the shear moduli and strength of wood. The existing literature does not provide a universal consensus over the best method for shear characterisation, although the existing test standards suggest using four-point bending and three-point bending tests. The four-point bending test configuration is depicted in Figure 4 [68].

Beliem et al. [69] analysed the effects of drying temperature and of the pressing time and temperature of wooden PF single-lap joints. For the drying stage, the joints were kept at 60 °C and 95 °C for four hours, and drying was analysed throughout this time. A temperature of 60 °C had no effect on the joint strength, while that of 95 °C showed degradation in the strength of the joint subjected to prolonged temperature exposure. Regarding the pressing parameters, 120 and 200 °C proved to be suitable for good adhesion; however, the increase in temperature led to a reduction in the hardness of the impregnated wood, which is believed to be promoted by hemicellulose degradation.

### 3.3. Wood Toughening Procedures

In both a composite laminate and a wood structure, premature failure often occurs in the form of delamination. Toughening methods are being developed to improve the transverse strength and, thus, the delamination resistance of those materials [70].

Brittle substrates generally lead to high levels of stress concentration in high-performance structures. Using stiff plies at the outer layers of composite laminates could effectively reduce the level of peel stress in modern composite and adhesive joint design [71]. Using a tough layer is one of the simpler and most effective methods to increase the transverse strength of laminated structures by reducing the peel stress level. In this method, a thin layer from a tough material is used at the surfaces of the laminated structures, where the peel stress is at the highest level compared to other areas through the thickness of the substrate. Therefore, the transverse strength of the entire structure is improved, and delamination is delayed or avoided. Under ideal conditions, the failure mode will change from delamination in the laminated structure to cohesive failure in the adhesive.

Using a metal laminate as a toughening layer was suggested for the first time in 1983 by Schijve et al. [72]. The authors reported that the key goal during the manufacturing process is to create a good adhesion between metal laminates and composite plies. New thermoplastic plies are another suggested technique to improve the transverse strength of wood-based substrates.

Another effective design factor is the variation of the thermal coefficient. There have been several research attempts on the general behaviour of fibre metal laminates (FMLs) [73]. Bano et al. [74] investigated the effects of the adhesive fillets and the geometrical parameters of the joints by comparing them with a reference joint made of CFRP substrates. A clear comparison between the reference CFRP and the FMLs is, however, missing in that work. Palmares [75] and dos Santos [76] investigated the configuration of adhesive joints with FML substrates. The experiments demonstrated that the metal ply results in a failure mode change from the substrate delamination in CFRP joints to cohesive failure within the adhesive layer in the FML joints. Their numerical study showed a reduction in the peel stress level and an improvement in joint strength by approximately 10%.

The improvements of the CFRP substrate by using thermoset and thermoplastic composite laminate in single-lap joints were considered by Shang et al. [77]. The authors suggested new toughened composite laminates using a thin layer of tough adhesive reinforced with glass fibres. The polymeric layer showed plastic deformation at the bondline surfaces, which caused a 22% improvement in the failure load. Furthermore, and perhaps most importantly, the failure mode changed from substrate delamination to cohesive failure within the adhesive layer.

### 3.4. Wood Delignification and Densification Methods

Wood is not a common choice for application in safety-critical structures because its mechanical properties are not high enough, and its natural defects make its mechanical response unpredictable. As a matter of fact, these two major limitations of wood hinder its use in high-performance structures [66,78]. To improve wood performance and expand its structural uses, such as in structural adhesive joints, one possible method is wood densification.

In recent decades, many methods have been proposed to increase the density and mechanical properties of wood, most of which are thermo–hydro–mechanical (THM) treatments. These methods are based on a number of hypotheses. Firstly, due to the viscoelastic nature of wood, its mechanical behaviour changes with time and environmental moisture [79]. Secondly, since the mechanical properties of lignin and hemicellulose change significantly with service temperature, the behaviour of wood also depends on temperature. As with most amorphous polymers, the behaviour of lignin and hemicellulose is affected by the *T*_g_. Above *T*_g_, these materials are “rubbery” and compliant, while the same materials are “glassy” and stiff below *T*_g_. The *T*_g_ of dry lignin has a value between 134–235 °C, and that of hemicellulose is between 167–217 °C. Although these values are generally not concerning, the *T*_g_ was seen to drastically decrease with an increasing moisture content [80]. It is known that moisture acts as a plasticiser that decreases the secondary bond between polymer chains. Therefore, the polymer molecules have higher flexibility to move [80]. 

Additionally, moisture causes the wood to swell. One of the main problems in the wood densification process is related to its irreversible swelling, which results from the moisture sensitivity of wood (known as set-recovery). Set-recovery will influence the absorbed energy in the calluses and covalent bonds between the polymeric chain. This sensitivity is attributed to the release of the elastic energy stored in the cellulose macromolecules and the unbroken covalent and hydrogen bonds between the polymers [80,81,82].

Sadatnezhad et al. [66] increased wood density using THM treatments. The set-recovery of the densified wood after three cycles of wet-drying was about 44%. A 60% set-recovery was reported by Laine et al. [83] after water-saturating densified specimens. However, this was almost eliminated by the application of a thermal modification treatment after the compression [84].

To mitigate the above issue while increasing the level of densification, chemical pre-treatments have been used [79]. These treatments are based on the delignification process, which is common in the paper industry, to expose the cellulose microfibrils, which are involved in lignin and hemicellulose. These methods lead to the partial or complete removal of lignin and, inevitably, hemicellulose. To achieve this purpose, a boiling alkaline media is usually employed to induce complex reactions, which result in the breakage of lignin-hemicellulose bonds and the degradation and solubilisation of the two materials. This process also improves the hydrophilicity of the material because lignin is the most hydrophobic component of wood, and therefore, when it is removed, cell wall accessibility is improved for water uptake. At the end of this process, the material demonstrates more porosity and is more flexible and formable. However, the cell wall structure is maintained and can be compressed without defects due to the reduced transverse stiffness of these walls due to the lignin removal [66,85].

The free OH groups enable the establishment of H-bonds between the cellulose fibrils when drying under compression, which allows densified wood to maintain its integrity while improving its mechanical properties, even without the lignin matrix. This is also supported by the physical interlocking of the cellulose fibres [85].

**Figure 5:**
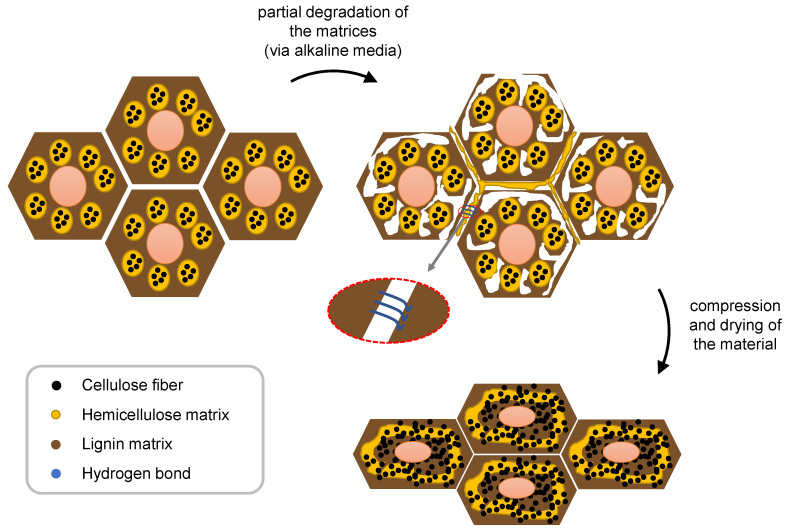
A scheme of the wood delignification and densification processes.

Figure 5 presents a scheme of the delignification and densification processes described above.

Methods that follow these general principles have been experimentally employed. Song et al. [86] boiled wood in a NaOH/Na_2_SO_3_ solution and then pressed it at high temperature. This procedure was applied to several softwood specimens in the radial direction and resulted in a strength of about 500 MPa, which corresponds to a very high specific strength, higher than for most structural alloys (422.2 ± 36.3 MPa cm^3^ g^−1^). Different degrees of delignification were analysed by changing the boiling times. It was seen that the higher density corresponded to the removal of 45% lignin. The densified specimens were seen to increase their thickness by 8% after 128 h at 95% RH, which led to a decrease in tensile strength of 16%. These are favourable values, especially when compared to natural wood. The chemical treatment was also seen to reduce the number of gaps left in the material after compression.

Novel et al. [87] performed a similar chemical treatment but added silicon carbide and graphene oxide particles to the specimens before pressing. These particles were reported to increase stiffness and tensile and flexural strength. The particle addition also contributed to a lower water uptake of the wood. Shi et al. [63] followed a similar procedure, achieving 80% wood densification in the radial direction. The tensile strength was reported to almost double, and the Young’s modulus was increased by nearly 350%.

Corte-Real et al. [14] conducted a delignification and densification process very similar to that proposed by Song et al. [86]. The authors reported that, during the densification process, an increase in the size of the defects naturally found in wood occurred. Therefore, the authors proposed that wood compression should always be performed inside a mould that promotes isostatic pressure.

In summary, wood substrates are increasingly becoming an alternative for structural applications due to their availability in nature and versatility in different applications. However, two main aspects should be considered when using wood. The first is the anisotropic mechanical behaviour of the material; as a result, care must be taken in the design process to take advantage of the grain direction of the wood. The second is related to the existence of natural defects in the wood structure. To overcome the setbacks of wood, innovative methods such as delignification and densification can be used. These techniques promise to reduce the effects of defects on the final structure and enhance the mechanical properties of wood.

## 4. Wood/Bio-Adhesive Joints

The joining of wooden components is traditionally carried out using mechanical fasteners such as nails, bolts, and screws [88,89,90]. However, these joining methods rely on the introduction of a hole to the substrates to be bonded. This causes a disruption in the wood fibres and creates a stress concentration site which, if not carefully selected, can lead to the macro-scale failure of the structure. In addition, introducing a foreign object into the wood’s structure can create a preferential path for water diffusion [91]. Joining wood using adhesives is a promising alternative joining method which will be discussed in this section.

### 4.1. Applications of Wood/Bio-Adhesive Joints

**Figure 6:**
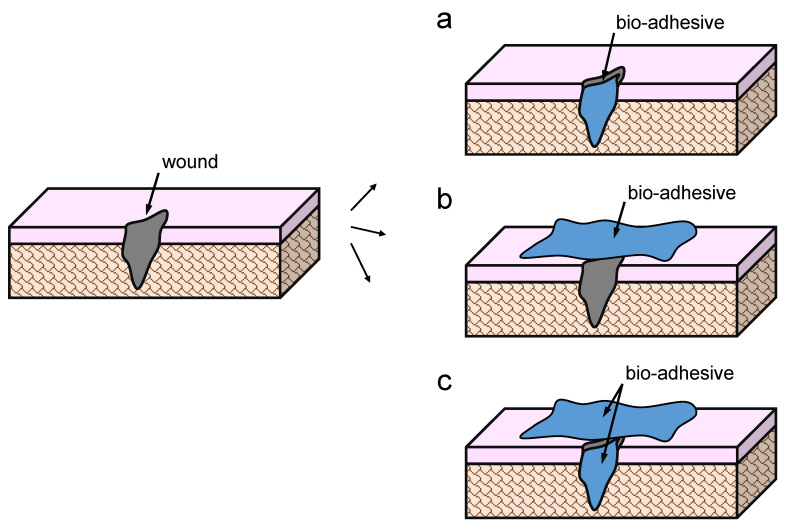
Bio-adhesives used for wound closure by (**a**) joining the tissue beneath the surface, (**b**) joining the two sides of the injury, or (**c**) combining both (adapted from [92]).

One of the most common applications of bio-adhesives is wound closure. Traditionally, sutures or staples are applied, even though they may lead to secondary injury or scar tissue [92,93]. Therefore, adhesives have been used as a non-invasive method, either by joining the tissue beneath the surface, joining the two sides of the wound, or a combination of both (Figure 6). The adhesives used in this application are carefully selected to be both biodegradable and biocompatible. In this specific application, it is particularly important that the adhesive can naturally degrade with time, so it will disappear from the wound and leave it healed [94].

Adhesives can also be used to join wood panels or beams in a structure. In fact, at this stage, adhesive bonding is presented as the most crucial process in the efficient use of wood structures. For this purpose, different adhesive families have been used, considering both synthetic and eco-friendly adhesives. The latter are preferential due to their lower impact on the environment. In all cases, the bondability of wood has been known to change with its surface properties, porosity, density, moisture content, and dimensional movement [95,96,97]. The easier wood to bond is the one with a lower density since the adhesive penetrates the wood better, leading to better mechanical interlocking.

Adhesives have been used to bond wood in a broad range of applications, from non- structural to structural ones, with the adhesive chosen to change for each particular application. 

The other critical categorisation of adhesive applications is if they are used in interior or exterior applications (i.e. if they can withstand environmental conditions). For non-structural interior applications, animal-based, soybean, and starch adhesives are already considered. The same stands for structural interior applications, where casein-based and blood-based adhesives can be suggested. However, for structural exterior applications, where adverse environmental conditions prevail, such as water-uptake cycles, high temperature, and radiation, petroleum-based adhesives are still preferentially used [95]. Therefore, research on wood/adhesive combinations, particularly those that can withstand severe environmental conditions, has been a topic of continuous research.

### 4.2. Mechanical and Fracture Toughness Characterisation of Wood/Adhesive Joints

To evaluate the mechanical and fracture toughness properties of an adhesive joint, two types of tests are generally used: strength and fracture tests. In wood joints, data post-processing is critical for evaluating the performance of the joint, especially the wood failure percentage (WFP) value [42]. This value is defined as the percentage of the area of the overlap that failed on the wood substrate. The higher the WFP, the stronger the bond is, given that the adhesive layer was not the main failure region. In the limit, if the failure occurs entirely on wood, the limit of the structure coincides with the limit of the substrate, which is the ideal situation for an adhesive joint. The two sub-sections below discuss not only bio-adhesives but also adhesives in general.

#### 4.2.1. Strength Tests

**Figure 7:**
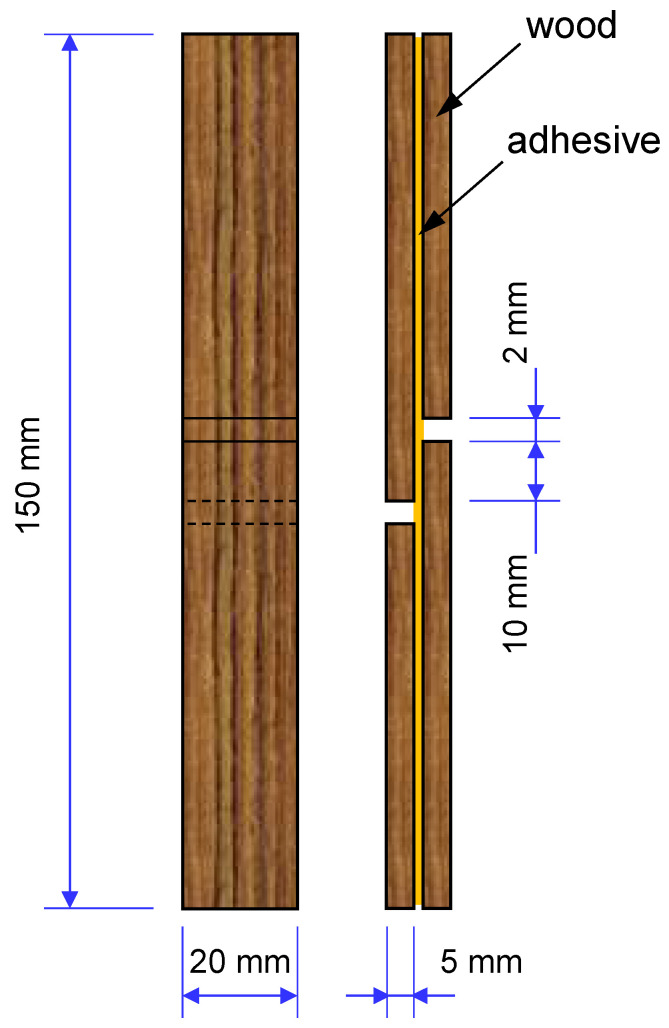
A scheme of the test specimen for determining the strength of wood adhesive joints according to EN 302-1 (2013).

The most common test method to evaluate the strength of an adhesive joint is the single-lap joint test. These joints are not used for the characterisation of the adhesive or substrate independently but to characterise the combination of both materials [1]. These joints have been widely used by academia and industry to characterise a wide variety of materials. For wood adhesive joints, in particular, single-lap joint testing also represents a preferential test method (Figure 7). The variety of conditions that can be evaluated from this type of joint is vast, from the adhesive/substrate bondability to the joint behaviour at different temperatures, humidity conditions, and loads, among others.

Müler et al. [98] produced single-lap joints using two adhesives: phenol–resorcinol–formaldehyde (PRF) and one-component PUR. An electronic digital image correlation method was used to measure the displacement fields in the joint. It was empirically seen that the ends of the overlap of the joint are highly stressed, which is consistent with the peaks in the tensile and shear stresses in this region.

Fecht et al. [99] studied hardwood single-lap joints under room temperature and high temperatures using experiments and numerical modelling. The authors showed that the strength of the joint significantly decreases as temperature increases. At 120 °C, the strength was about 40% of the strength at 22 °C. At room temperature, the failure of the joint is governed by the failure of wood, and it can be predicted probabilistically. The prediction remains valid up to 120 °C, at which temperature it becomes inaccurate, showing that further characterisation is needed for high temperatures. In high temperatures, the failure strength of the joint showed a significant reduction (of about 85%) because of the adhesive and substrate degradation caused by the drying of the wood structure.

Clauß et al. [100] studied the thermal stability of wood adhesive joints using single lap joints. The joints were manufactured using different adhesives and tested from 20 °C to 250 °C. As the temperature increased, the adhesive joint lost some of its strength, while the same trend was also observed for the wood substrate. In addition, different adhesives exhibited different sensitivities to temperature.

Tannert et al. [101] investigated the effect of overlap length on the strength of wood adhesive joints. Double-lap joints with overlaps of 80 to 320 mm were tested under static conditions. The results showed that the joint strength and the overlap length have no direct correlation. It was also observed that the failure begins with a combination of tensile and shear stresses at the ends of the overlap, particularly at the adhesive interface.

Tiryaki et al. [102] used these joints to study the effects of adhesive amounts, pressing temperature, and pressing time on the bonding strength of a wood joint with a polyvinyl acetate adhesive. An artificial neural network model was developed to predict joint failure. Increasing the amount of adhesive, the pressing temperature, and the pressing time positively affected joint strength. The neural network developed also showed encouraging results, with an error of 2.5% and a correlation coefficient of 98%.

Corte-Real et al. [14] tested single-lap joints with wood substrates, wood substrates reinforced with cork, and densified wood substrates. In addition, quasi-static, intermediate, and impact loading speeds were considered. The authors found that the energy absorption at high strain rates could be increased using specially modified substrates.

#### 4.2.2. Fracture Tests

**Figure 8:**
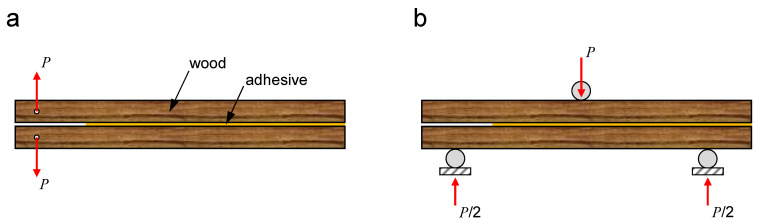
Simplified schemes of the (**a**) DCB and (**b**) ENF test configurations for studying the fracture behaviour of wood adhesive joints.

The most common fracture tests for adhesive joints in general, including wood adhesive joints, are the double cantilever beam (DCB) and end-notched flexure (ENF) tests (Figure 8). These tests are performed for adhesive characterisation and determination of the mode I and mode II fracture toughnesses of the joint.

Xavier et al. [103] used wood joints with an epoxy adhesive and tested them in mode I and mode II loadings using DCB and ENF tests, respectively. The data for both cases were treated using the compliance-based beam method (CBBM). This method is known to be independent of direct crack length measurement, which is particularly practical for wood substrates in which crack measurement is often ambiguous. The CBBM method was validated for the joints used.

Silva et al. [104] tested wood adhesive joints using the ENF test and determined the strain cohesive law of bonded joints, which exhibited a good agreement between the direct and inverse methods. This shows that appropriate results can be obtained using the inverse method.

Gaspard Clerc et al. [64] tested wood adhesive joints by employing the four-point ENF test under quasi-static and fatigue loading conditions. Three adhesive types were considered: one brittle PRF and two one-component PURs. All three adhesives had similar energy release rate values under quasi-static conditions. Under fatigue loading, however, the more brittle adhesive demonstrated slower crack growth than the other two. The study showed that the wood adhesive joints had satisfactory performance, and the adhesive/substrate interface was strong because cohesive failure occurred.

In a more general sense, wood adhesive joints are basically used to characterise the adhesive used to bond wood. However, these joints can sometimes be more useable, allowing for an understanding of the relationship between the fracture toughness of the wood and adhesive or of the quality of the bond.

### 4.3. Effects of Moisture

A wood adhesive joint often needs to operate in a high-moisture environment. Moisture absorption causes wood to shrink or swell and, consequently, pre-stresses the adhesive layer. Since the moisture content of wood changes continuously with the environmental conditions to which it is exposed, moisture can affect the strength of the final joint. It may lead to wood delamination or even to the macro-scale failure of the whole joint in extreme cases.

The effect of increased moisture on the mechanical response of wood adhesive lap joints was studied in [105,106] under quasi-static loading. Five different adhesives that are commonly employed in industrial applications of wood were studied: two one-component moisture-curing PUR adhesives, one PRF resin, and one melamine–urea–formaldehyde (MUF) resin. Using adhesive films, the authors investigated how RH affects adhesive strength and Young’s modulus. Furthermore, two non-commercial 1C-PUR pre-polymers were used, each with a specific proportion of ethylene oxide (EO). The tensile tests performed showed that for all tested adhesives except PRF, the tensile strength and Young’s modulus were both linearly dependent on the RH; increasing the RH significantly decreases both these properties. It was also observed that, for all adhesive types, as the wood moisture content increases, the shear strength decreases.

Ammann and Niemz [107,108] investigated the effect of moisture on the fracture properties of wood adhesive joints. In their first study [107], the authors used the Arcan test setup and examined five fracture mode angles, ranging from pure mode I to pure mode II. The effect of moisture was taken into consideration by manufacturing specimens at 50%, 65%, and 90% RH. As expected, the fracture toughness parameter, *K*_c_, increased as mode II stress levels were increased. In response to changes in RH, the rate of increase changed. The authors also found that the adhesive type has a clear effect on the fracture toughness of joints. Adhesives become more ductile through exposure to moisture.

In their subsequent study [108], the same authors used wooden DCB specimens to study the effect of moisture on fracture energy. Adhesives were bonded under the same RH conditions, using two adhesives: PUR and PRF resin. Due to the low fracture energy of the first adhesive, moisture had a negligible effect on the respective specimens. In PRF-bonded specimens, in contrast, the effects of moisture were noticeable. It was estimated that energy absorption during the test increases with increasing humidity because the joints are more ductile. In most cases, adhesion and delamination were the main causes of failure.

Hassani et al. [109] performed an experimental and numerical investigation of the effect of moisture on the fracture performance of adhesively bonded laminated wood. More specifically, the effects of real changes in climate on the delamination and fracture of hardwood adhesive joints were investigated. European beech panels were produced with three plies, either aligned or crosswise, which were bonded together using PUR, PRF, and MUF adhesives. Various laminate thicknesses were examined, from 8 mm to 60 mm. The grain direction of the middle ply was perpendicular to that of the outer plies, and the middle ply was twice as thick as the outer plies. An RH of 95% was maintained, and the specimens were prepared under cyclic RH conditions. After drying, each specimen was wetted to 95% RH. Each specimen was subjected to these cycles three times. After drying, the specimens were measured in all dimensions. In addition, possible intralaminar or interlaminar damages were monitored after varying numbers of cycles. According to the fracture surfaces obtained, PUR and PRF joints failed predominantly by interphase failure, while MUF joints failed primarily by adhesive failure. Due to the higher fracture toughness of PUR and PRF adhesives, the respective bonded joints displayed superior performance, leading to substrate degradation and failure. As a result of the gradient of moisture near the adhesive bondline, the substrate was deformed, and local stress concentrations were generated along the bondline. This damaged the adhesive.

For moisture investigation, it is critical to measure the exact strain field at the crack tip, which is the initiation point of delamination. In addition to deformation values, the modelling of moisture effects requires deformation values. A spatially resolved approach was found to be necessary to investigate wood joint delamination and water absorption. Using neutron radiography, it is possible to quantify and locate moisture within wood specimens [110]. 

As part of their study, Sonderegger et al. [111] analysed neutron radiographs of wood adhesive joints using three different adhesives. Joints were manufactured taking the grain direction into consideration. A study of the moisture content and diffusion coefficients in the joints was conducted by the authors. According to reports, neutron radiography has many limitations, including time constraints. As an example, obtaining experimental results is time-consuming in some cases because water uptake and diffusion in the joints can take several months. In addition, the neutron imaging facility is limited by beam times, making it impossible to reliably accomplish this. To minimise the diffusion coefficient, it is essential to oven-dry the specimens before imaging the reference joints. As a result of the drying procedure, the micro-structure of wood can be affected by thermal modifications, which, in turn, are affected by the sorption behaviour of wood [112]. In addition, the specimens resorb water (3–5%) during preparation and handling, preventing them from achieving the desired dry reference state [113]. As a result of the moisture-induced swelling associated with neutron measurement, specimens imaged in different states may be misaligned. Due to this, the calculated moisture maps contain strong artefacts and discontinuities (glue lines and outer edges) [113]. Additionally, swelling strain and neutron transmission affect the local moisture concentration. Calculating moisture values without accounting for the aforementioned factors leads to quantitative uncertainties. It is, therefore, necessary to determine deformation fields so that moisture can be quantified and localised accurately.

Using a VIC 3D system (optical image correlation) in combination with neutron imaging, Lanvermann et al. [114] addressed this problem. A speckle pattern is continuously recorded on the coupon by two CCD cameras. By calculating surface deformation fields with high spatial resolution using the acquired data, the specimen surface deformation fields are determined. Despite its spatial accuracy, this setup is complex, and its primary use is in determining how the heterogeneous wood microstructure influences moisture transport. Wood substrates are generally considered to be homogeneous layers for the purpose of diffusion analysis across adhesive bonding. When this is the case, it is desirable to use neutron radiographs exclusively for the correction of deformation.

### 4.4. Effects of Fatigue Loading

Structures can suffer premature failure over time if long-term cyclic loads accumulate at stresses below the static strength of the material [115]. Wooden structures are highly susceptible to fatigue loading. The fatigue of wood was, in fact, an active research topic until the Second World War because the wood was often used in load-bearing structures in construction and other sectors at that time. Then, the research focus shifted towards composite materials, thanks to their advantageous mechanical properties and low weight [116,117]. Today, wood is used in both traditional and advanced structures. Hence, the mechanical behaviour of wood adhesive joints and the factors affecting their durability should be well investigated. Various researchers have studied these parameters under quasi-static loading conditions [118,119], and recent works have investigated different fatigue loading conditions in wood joints.

Bachtiar et al. [120] investigated the shear behaviour of wood adhesive lap joints using three brittle adhesives (namely, MUF, fish glue, and bone glue adhesives) and one ductile adhesive (namely, PUR). The moisture content of all specimens was about 11% under climatic conditions (20 °C and 65% RH), which is a crucial parameter that directly affects the mechanical performance of the joints and should be controlled during manufacturing and testing. Joint behaviour does not change significantly when the adhesive properties are quasi-static. It was evident that adhesive effects were present in fatigue. A variety of adhesive moduli were responsible for determining fatigue behaviour in the fatigue region. High-intensity loads and low-cycle fatigue seem to be the strongest conditions for joints with brittle adhesives. Meanwhile, joints bonded with PUR could dampen lower loads perfectly and perform well under high-fatigue and low-intensity conditions. It can be concluded that PUR converts larger amounts of strain energy into heat compared to the other adhesives. As a result, joints have a longer fatigue life, thereby delaying damage accumulation in the joints [121].

It is more common for wood joints bonded with brittle adhesives to fail. The primary reason for this is the higher peel and shear stress concentrations at the end of the bondline. Although adhesive systems exhibit different adhesion phenomena based on their chemical nature, brittle-tested adhesives generally possess a densely interconnected polymer network, resulting in a higher Young’s modulus and providing more connecting points to the wood, which, in turn, offers better adhesion and a higher WFP [120].

## 5. Conclusions

Wood/bio-adhesive joints are gaining importance in several industrial fields due to their eco-friendly properties and ability to withstand significant loads. This paper reviewed adhesive joints consisting of bio-adhesives and wood-based substrates, highlighting recent methods to improve these constituent materials, accurately characterise them, and implement them in adhesive joints.

The main conclusions that can be drawn from this review are summarised below:

- Bio-adhesives are steadily developing to gain properties similar to those of synthetic materials. Tannin, lignin, carbohydrates, proteins, and other biomaterials have been proposed as a base for these adhesives. Most of these materials must undergo adaptations before they are used industrially. However, in terms of mechanical properties, they are very promising. At this stage, the main concerns are related to the resistance of these materials to harsh environments, such as high temperatures and humidities.
- Wood has the important advantage that it can be machined into different forms. However, in its neat form, it presents limited strength and stiffness for safety-critical structural applications. Additionally, natural defects in this material may lead to structural failure. There are methods under development related to the delignification and densification of wood, which can reduce the impact of defects and make the properties of wood close to those of conventional composites used in the automotive or aerospace industry.
- Substrate toughening, paired with densification, may be an interesting alternative to improve the mechanical behaviour of wood/bio-adhesive joints by reducing the stress level in the substrate at the ends of the overlap. Only a few toughening methods using biomaterials are now available for these joints, but this may be a promising path for future research.
- Wood/bio-adhesive joints have been proven to be applicable to a wide range of structures, and the influence of moisture and fatigue on their properties has been investigated. It was seen that this is still a setback of natural joints since both wood and bio-adhesives are sensitive to humidity, temperature, and fatigue loading conditions. Thus, characterisation and development work should be carried out on this topic.

## Data Availability

Not applicable.
